# Calciphylaxis: ongoing challenges and treatment opportunities with mesenchymal stem cells

**DOI:** 10.1093/jmcb/mjaf009

**Published:** 2025-03-17

**Authors:** Daoxu Wu, Shijiu Lu, Jiaying Hu, Ming Zeng, Jingjing Wu, Cui Li, Xingfang Tang, Tian Lu, Yi Zhu, Jiayin Liu, Lianju Qin, Ningning Wang

**Affiliations:** Division of Nephrology, Department of Geriatrics, the First Affiliated Hospital with Nanjing Medical University, Jiangsu Province Hospital, Nanjing 210029, China; Department of Nephrology, the First Affiliated Hospital with Nanjing Medical University, Jiangsu Province Hospital, Nanjing 210029, China; Department of Nephrology, Yantai Yuhuangding Hospital, Yantai 264000, China; Division of Nephrology, Department of Geriatrics, the First Affiliated Hospital with Nanjing Medical University, Jiangsu Province Hospital, Nanjing 210029, China; Department of Nephrology, the First Affiliated Hospital with Nanjing Medical University, Jiangsu Province Hospital, Nanjing 210029, China; Division of Nephrology, Department of Geriatrics, the First Affiliated Hospital with Nanjing Medical University, Jiangsu Province Hospital, Nanjing 210029, China; Department of Nephrology, the First Affiliated Hospital with Nanjing Medical University, Jiangsu Province Hospital, Nanjing 210029, China; Department of Nephrology, the First Affiliated Hospital with Nanjing Medical University, Jiangsu Province Hospital, Nanjing 210029, China; Department of Nephrology, the First Affiliated Hospital with Nanjing Medical University, Jiangsu Province Hospital, Nanjing 210029, China; Division of Nephrology, Department of Geriatrics, the First Affiliated Hospital with Nanjing Medical University, Jiangsu Province Hospital, Nanjing 210029, China; Department of Nephrology, the First Affiliated Hospital with Nanjing Medical University, Jiangsu Province Hospital, Nanjing 210029, China; Department of Nephrology, Nanjing Pukou People's Hospital, Nanjing 211800, China; Division of Nephrology, Department of Geriatrics, the First Affiliated Hospital with Nanjing Medical University, Jiangsu Province Hospital, Nanjing 210029, China; Department of Nephrology, the First Affiliated Hospital with Nanjing Medical University, Jiangsu Province Hospital, Nanjing 210029, China; The Affiliated Lianyungang Municipal Oriental Hospital of Kangda College of Nanjing Medical University, Lianyungang 222042, China; School of Medicine, Westlake University, Hangzhou 310024, China; Westlake Center for Intelligent Proteomics, Westlake Laboratory of Life Sciences and Biomedicine, Hangzhou 310024, China; School of Medicine, Westlake University, Hangzhou 310024, China; Westlake Center for Intelligent Proteomics, Westlake Laboratory of Life Sciences and Biomedicine, Hangzhou 310024, China; State Key Laboratory of Reproductive Medicine and Offspring Health, Center of Clinical Reproductive Medicine, the First Affiliated Hospital with Nanjing Medical University, Nanjing 210036, China; State Key Laboratory of Reproductive Medicine and Offspring Health, Center of Clinical Reproductive Medicine, the First Affiliated Hospital with Nanjing Medical University, Nanjing 210036, China; Division of Nephrology, Department of Geriatrics, the First Affiliated Hospital with Nanjing Medical University, Jiangsu Province Hospital, Nanjing 210029, China; Department of Nephrology, the First Affiliated Hospital with Nanjing Medical University, Jiangsu Province Hospital, Nanjing 210029, China; Jiangsu Provincial Key Laboratory of Biological Therapy for Organ Failure, Nanjing Medical University, Nanjing 211166, China

**Keywords:** calciphylaxis, calcific uremic arteriolopathy, orphan disease, microvascular calcification, microthrombus, infection, mesenchymal stem cells, biomarkers

## Abstract

Calciphylaxis is a rare, progressive disorder characterized by subcutaneous adipose and dermal microvascular calcifications, microthrombi, and endothelial damage. It mainly affects patients with chronic kidney disease (CKD), which is also known as calcific uremic arteriolopathy. Skin biopsy is the gold standard for diagnosis, but it is an invasive procedure. Calciphylaxis frequently results in ischemic and nonhealing ulcerations with a high mortality rate. A multidisciplinary targeted approach is the primary treatment method. Vascular calcification, which is a common complication in patients with CKD, cannot completely explain the rapid progression of calciphylaxis. This article reviews the advances in the epidemiological characteristics, risk factors, and diagnosis, including non-uremic calciphylaxis and visceral calciphylaxis, pathogenesis, associated animal models, and treatment of calciphylaxis. The scarcity of animal models that mimic the clinical presentation of calciphylaxis hampers the understanding of its pathogenesis. The acute effects on progressive vascular injury, including the induction of severe ischemia and inflammatory responses, have been emphasized. Actively listening to the voices of patients and their families and building a multidimensional research system with artificial intelligence technologies based on the specific molecular makeup of calciphylaxis patients will help tailor regenerative treatment strategies. Mesenchymal stem cells (MSCs) may represent a novel therapy for calciphylaxis because of their regenerative effects, inhibition of vascular calcification, anti-infection and immunomodulation properties, and improvement of hypercoagulability. Safe, effective, accessible, and economical MSC strategies guided by biomarkers deserve consideration for the treatment of this devastating disease.

## Introduction

Calciphylaxis, also known by the International Classification of Diseases, 10th Revision (ICD-10) diagnosis code E83.5, is a rare dermatological disorder classified as an orphan disease (ORPHA: 280062). It is characterized by calcium deposition in the microvasculature of the dermis and subcutaneous adipose tissue, endothelial damage, fibrointimal proliferation, and microthrombus formation (Nigwekar et al., 2018; Rick et al., 2022b). It commonly occurs in patients with chronic kidney disease (CKD), especially those with end-stage kidney disease (ESKD), which is known as calcific uremic arteriolopathy (CUA), with an annual incidence rate of ∼0.04%–4% (Liu et al., 2022b). Non-uremic calciphylaxis (NUC) has also been reported. Yousuf et al. (2024) reported 21 patients with NUC and 39 patients with CUA at a single dermatological center in Germany over 10 years.

Patients with calciphylaxis typically have severe, painful cutaneous lesions and tactile hyperesthesia, with a median survival time of 3 months and a 1-year mortality rate of 80% due to ulceration and infection (Weenig et al., 2007; McCarthy et al., 2016; Nigwekar et al., 2018). Currently, there are no approved treatments or standardized guidelines, which poses a clinical challenge (Gallo Marin et al., 2023). Mesenchymal stem cells (MSCs) exhibit various biological effects such as inhibition of calcification, promotion of angiogenesis, anti-inflammatory and immunomodulatory effects, improvement of hypercoagulability, and tissue repair (Hade et al., 2021; Wei et al., 2021; Qin et al., 2022; Bian et al., 2023). Here, we provide an overview of the research advances in the field of calciphylaxis, including the diagnosis of NUC and visceral calciphylaxis, pathogenesis, associated animal models, and treatment. Furthermore, we discuss the potential of MSC therapy for this devastating illness.

## Epidemiological characteristics and risk factors of calciphylaxis

### Epidemiological characteristics of CUA

A large-scale national study conducted in the United States revealed that the incidence of CUA among patients receiving maintenance hemodialysis was 3.49 cases per 1000 patient-years (Nigwekar et al., 2016). Over the past decade, the incidence of CUA has increased in the population undergoing dialysis. In 24 hemodialysis centers in China, the incidence of CUA was reported to be 1.24% (Liu et al., 2022b). The Mayo Clinic reported a 6-month survival rate of 57% among 101 patients with calciphylaxis (McCarthy et al., 2016). A retrospective study about the prognosis of CUA revealed a 6-month mortality rate of 37.2% and a 1-year mortality rate of 44.1%, and age and ESKD were identified as risk factors for 1-year mortality (Gabel et al., 2021b). Recently, in the Australia and New Zealand Dialysis and Transplant Registry (ANZDATA), calciphylaxis has been identified as a life-threatening condition in dialysis patients, with the greatest mortality burden within 12 months since diagnosis (Toussaint et al., 2024).

### Epidemiological characteristics of NUC

NUC has been reported in non-ESKD patients, including those with mild kidney dysfunction, acute kidney injury, or normal kidney function (Shahzad et al., 2021; Ababneh et al., 2022), as well as in those suffering from connective tissue diseases (Zhou et al., 2023). A systematic review of case reports and case series of NUC encompassed 36 cases (75% female, aged 15–82 years) (Nigwekar et al., 2008). Mineral abnormalities were often absent as potential causes, indicating different mechanisms of pathogenesis. The overall mortality rate was 52%. Most of the deaths occurred between 2 weeks and 1 year after the diagnosis, and sepsis was the leading cause. Epidemiological differences were not significant between the CUA and NUC patients in a study conducted at a single dermatological center in Germany (Yousuf et al., 2024).

### Risk factors of calciphylaxis

Risk factors for calciphylaxis include ESKD, diabetes, obesity, female gender, peritoneal dialysis, hypercoagulable state, hypercalcemia, hyperphosphatemia, secondary hyperparathyroidism (SHPT), vitamin D deficiency, polymorphism of vitamin D receptor, hypoalbuminemia, use of vitamin K antagonists (VKA) or vitamin K deficiency, iron therapy, repetitive local trauma, and thrombophilia (such as deficiency of antithrombin, protein C deficiency, or lupus anticoagulants) (Hayashi et al., 2012; Nigwekar et al., 2016, 2018; Rothe et al., 2017; Colboc et al., 2019; Seethapathy and Noureddine, 2019; Gallo Marin et al., 2023).

A cohort study showed that 60% of patients with CUA had a predisposition to severe thrombosis, and elevated plasma fibrinogen and D-dimer levels were present in 47% and 41% of patients, respectively (El-Azhary et al., 2016). A case–control study involving 38 patients with CUA and 114 patients with CKD showed that lupus anticoagulant, protein C deficiency, and comorbid susceptibility to thrombosis were all associated with CUA (Dobry et al., 2018). Brandenburg et al. (2017) showed that ∼50% of CUA patients in a German registry used VKA. Liang et al. (2023) reported a case of a 72-year-old woman with CUA who had undergone mechanical valve replacement and received warfarin treatment. The early use of direct oral anticoagulants (DOACs) may be beneficial in reducing the incidence of calciphylaxis (Liang et al., 2023).

**Figure 1:**
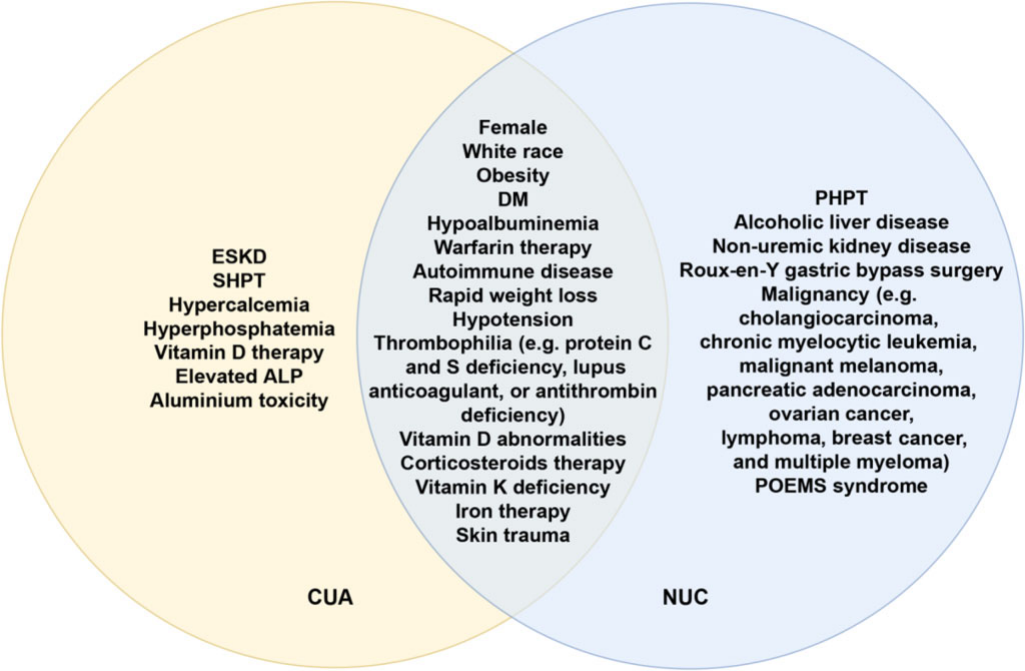
Comparison of potential risk factors for CUA and NUC. ALP, alkaline phosphatase; DM, diabetes mellitus; POEMS, polyneuropathy, organomegaly, endocrinopathy, M-protein, and skin changes.

The associated comorbidities in NUC mainly include primary hyperparathyroidism (PHPT), malignancy, alcoholic liver disease, connective tissue disease, and corticosteroid use. However, mineral abnormalities present in CUA are often absent in NUC, suggesting the heterogeneous mechanisms between the two diseases (Nigwekar et al., 2008). In addition, there have been some case reports of NUC as a complication of Roux-en-Y gastric bypass surgery (Allegretti et al., 2014), Hodgkin lymphoma (Sibai et al., 2012), teriparatide treatment (Dominguez and Goldman, 2014), and hypoparathyroidism (Erdel et al., 2014). The comparison of potential risk factors for CUA and NUC is shown in Figure 1.

## Diagnostic criteria for calciphylaxis

The diagnosis of calciphylaxis currently relies on the typical characteristics of skin lesions and pathological features (Weenig et al., 2007; Nigwekar et al., 2018). Their insidious onset and diverse manifestations increase the risk of misdiagnosis and underdiagnosis. An observational study conducted at the Massachusetts General Hospital reported a misdiagnosis rate of 73.1% among 119 patients initially diagnosed with CUA (Gabel et al., 2021a). Among patients with ESKD, the probability of an initial misdiagnosis was lower than in individuals without this condition (Gabel et al., 2021a). The diagnostic criteria and evaluation considerations for NUC are almost the same as those for CUA (Nigwekar et al., 2015).

### Clinical characteristics of cutaneous lesions in calciphylaxis

**Figure 2:**
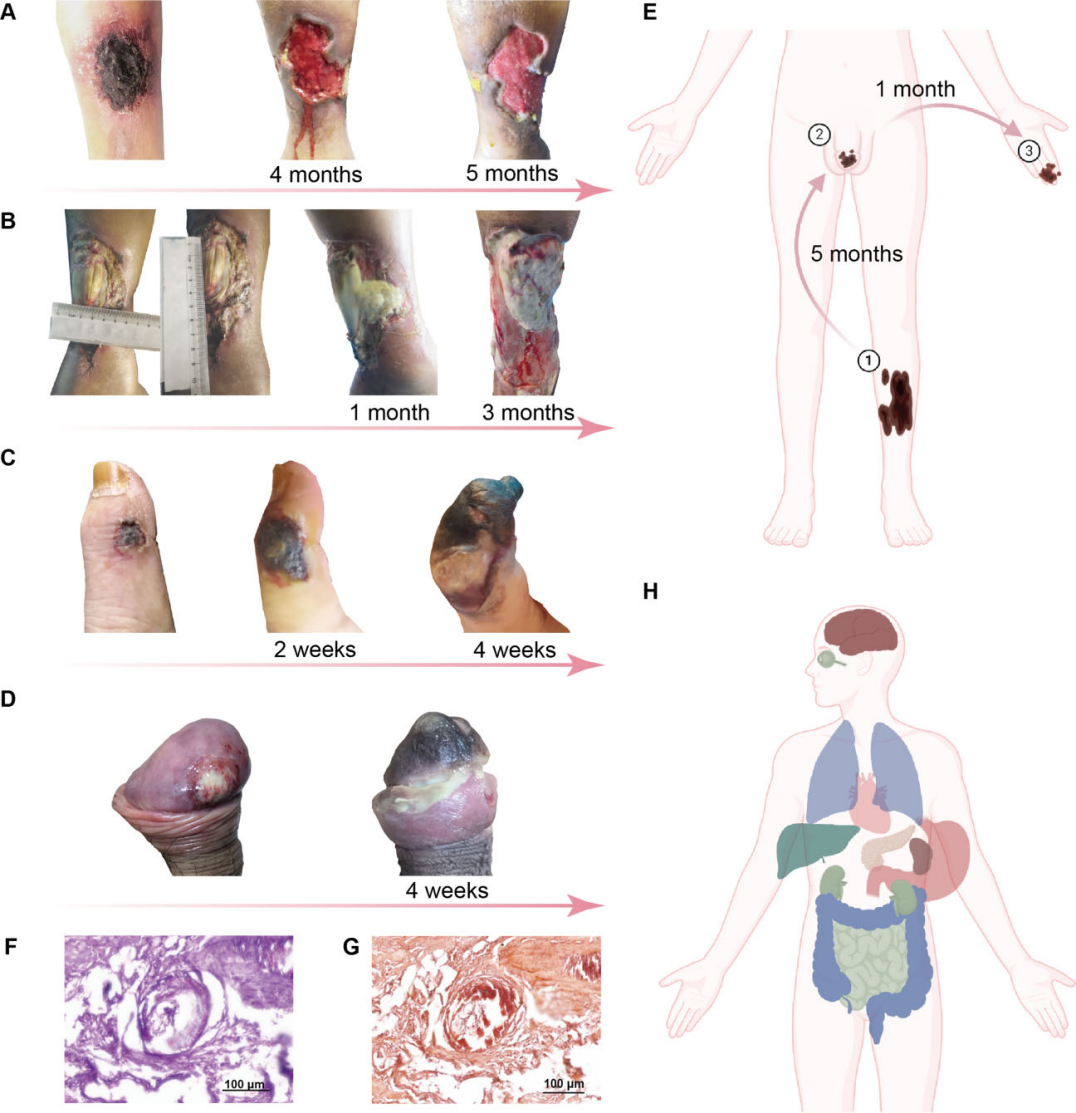
Dynamic skin lesion changes, typical pathological manifestations, and multiple internal organs are involved in patients with calciphylaxis. (**A**) Initial onset of black eschar on the side of the left leg developed an ulcerated surface after 4 months. The ulcer showed a reduction in size, with increased formation of granulation tissue, after application of conditioned medium from human amnion-derived MSC (hAMSC-CM). (**B**) The patient's left lower limb skin and soft tissue underwent necrosis with infection, exposing the Achilles tendon and emitting a foul odor. Within 3 months, the ulceration area gradually expanded and the lesion deepened, accompanied by purulent discharge and bleeding. (**C**) Initially, a black eschar, approximately the size of a soybean, appeared on the right hand fingers. Within 4 weeks, the area of necrosis rapidly expanded, accompanied by skin gangrene, swelling, and infection, affecting an area beyond the distal two phalanges. (**D**) The head of his penis initially presented with a white ulcer. Subsequently, he developed distal penile ischemia and gangrene within 4 weeks. (**E**) Overview of skin lesion progression in this patient with CUA. Skin lesions started on his leg. After 5 months, his penis was involved. Then, finger lesions also occurred within 1 month. (**F** and **G**) Skin pathological features in the patient with CUA. (**F**) Hematoxylin–eosin staining revealed local thrombus formation in the skin microvessels, along with suspicious calcium salt deposition in the vessel walls. (**G**) Alizarin red staining confirmed the presence of calcium salt deposition in the vessel walls. (**H**) The patient may exhibit calcification in multiple extracutaneous tissues, including the brain, optic nerve, lungs, heart, liver, pancreas, stomach, kidneys, spleen, mesentery, and colon.

In the initial phases of calciphylaxis, pain may occur prior to the formation of skin lesions, and its intensity may vary, often accompanied by increased tactile sensitivity (Ghosh et al., 2017). Early clinical manifestations of skin lesions include indurations, plaques, nodules, reticular or grapelike purpura, and ecchymoses (Weenig et al., 2007; Daudén and Oñate, 2008; Gallo Marin et al., 2023). Without effective treatment, the lesions can rapidly progress to malodorous ulcers accompanied by the formation of black eschar (Weenig et al., 2007; Daudén and Oñate, 2008). The Bates–Jensen Wound Assessment Tool-CUA (BWAT-CUA) is a modified tool specifically designed for CUA, which reduces the original 13 scoring items to 8. These include the necrotic tissue type, the amount of necrotic tissue, the exudate type, the amount of exudate, the surrounding skin color, the surrounding tissue edema, the surrounding tissue induration, and the granulation tissue. Each item is scored from 1 to 5, with a total score ranging from 8 (best) to 40 (worst) (Gould et al., 2021). Lesions in warfarin-associated calciphylaxis are usually located below the knees. Skin lesions in NUC can show both proximal and distal distributions, similar to CUA (Nigwekar et al., 2015). Approximately 50% of patients are unable to ambulate or require wheelchair assistance due to complications from skin damage, and over 70% of patients require hospitalization due to severe skin ulcers (Weenig et al., 2007). Figure 2A–E demonstrates the dynamic changes of skin ulcers in a patient with CUA.

### Pathological characteristics of cutaneous and visceral calciphylaxis

#### Cutaneous calciphylaxis

Skin biopsy is the gold standard for diagnosing calciphylaxis. It is recommended to perform a deep wedge biopsy, including subcutaneous tissue, and to conduct von Kossa staining (Cassius et al., 2018) or Alizarin Red staining for the calcification area (Bahrani et al., 2020). However, skin biopsy may potentially induce ulceration, infection, or the formation of new necrotic areas (Bahrani et al., 2020).

The key histopathological characteristics of calciphylaxis encompass intimal fibroplasia within the microvessels; extracellular deposition of calcium; calcification affecting the capillaries, internal elastic lamina, or subcutaneous glands; medial calcification occurring in subcutaneous arteries; arteriolar and arterial thrombosis in the dermis and subcutaneous tissue; and necrosis of skin (Weenig et al., 2007; Mochel et al., 2013; Chen et al., 2017). Among patients undergoing hemodialysis, the presence of vascular calcification within the interstitial tissue and/or subcutaneous tissue with a diameter of less than 500 μm indicates a strong likelihood of CUA (Christiadi and Singer, 2018). In a multicenter cohort study involving 36 patients, the calcification in calciphylaxis was primarily composed of pure calcium phosphate hydroxyapatite (HAP). It affected the intima but also extended to the media of the microvessels. It was commonly associated with interstitial deposits, which differ from the calcification observed in the vessels with atherosclerosis (Brouns et al., 2020). Figure 2F and G illustrates the typical pathological features of the skin in a patient with CUA.

*Visceral calciphylaxis.*  Andersen et al. (2014) reviewed autopsy reports of three CUA patients. All individuals exhibited histopathological evidence of cardiovascular calcification, as well as atherosclerosis affecting both the coronary arteries and the aorta. Calcification of pancreatic and renal vessels was also observed. However, there was no evidence of calciphylaxis in other organs. Therefore, the authors inferred that calciphylaxis only involves the skin and does not appear to involve extracutaneous organs (Andersen et al., 2014). However, this study had a small number of calciphylaxis patients, and it could have had selection bias. An increasing number of studies have demonstrated that calciphylaxis affects organs beyond the skin. Tan et al. (2020) reported a case of isolated mesenteric calciphylaxis in a hemodialysis patient, along with ischemic colitis, without active cutaneous calciphylaxis. This patient exhibited circumferential mural calcifications, as well as intimal/medial hyperplasia, resulting in the narrowing of the lumen in the mesenteric vessel (Tan et al., 2020). There have been other case series of biopsy-confirmed mesenteric calciphylaxis in hemodialysis patients, who presented with symptoms such as intestinal ulceration, bleeding, or ischemia (Tamura et al., 1995; Volpini and Kinonen, 2011). Besides mesenteric calciphylaxis, there is mounting evidence regarding the presence of calciphylaxis in internal organs, such as lung calciphylaxis and cardiac calciphylaxis (Tamura et al., 1995; Shiraishi et al., 2006; Alam et al., 2012; Kim et al., 2012; Stârcea et al., 2018; Opdebeeck et al., 2020). Figure 2H and Table 1 illustrate the various extracutaneous tissues that may be implicated in calciphylaxis.

**Table 1:** Calciphylaxis may involve calcification of multiple extracutaneous tissues.

Author (year)	Age (years)	Gender	Dialysis mode	Dialysis period (months)	Cutaneous involvement	Evidence for cutaneous involvement	Extracutaneous tissues involvement	Evidence for extracutaneous tissues involvement
Shi et al. (2021)	52	Female	HD	84	Yes	Skin biopsy	Lung	Lung biopsy, CT, and whole-body bone scan with ^99m^Tc-methylene diphosphonate
Tan et al. (2020)	40	Male	HD	2	No	NA	Penis and mesentery	Penile histopathologic examination, abdominal CT, and histologic examination of the resected bowel
Mitchell et al. (2020)	49	Male	HD	36	Yes	Skin biopsy	Colon	Abdominal CT and radiograph
Stârcea et al. (2018)	4	Male	PD	24	Yes	Autopsy	Brain, heart, lung, kidney, stomach, and pancreas	Autopsy
Arrestier et al. (2016)	49	Female	HD	60	Yes	Skin biopsy	Lung	Bronchial biopsy
Komurcu et al. (2016)	43	Female	HD	NA	Yes	Skin biopsy	Bilateral optic neuropathy, mesentery, spleen, and kidney	Doppler ultrasonography of ciliary arteries and abdominal CT
Gupta et al. (2015)	66	Female	HD	NA	Yes	Radiograph of the legs; no skin biopsy	Extensive vascular calcifications and bleeding in the gastrointestinal tract	Abdominal CT and colonoscopy; no biopsy
Kim et al. (2012)	40	Male	HD	1.5	Yes	Autopsy	Lung and kidney	Autopsy
Alam et al. (2012)	45	Male	HD	NA	Yes	Autopsy	Heart	Autopsy
Li et al. (2006)	25	Female	HD	1	No	NA	Lung	Chest radiography, high-resolution CT, and lung biopsy
Tamura et al. (1995)	53	Female	HD	NA	Yes	Autopsy	Cerebral, myocardial, splenic, and intestinal infarctions and calcification	Autopsy

HD, hemodialysis; PD, peritoneal dialysis; NA, not available.

### Imaging characteristics of calciphylaxis

When a skin biopsy is negative or when an invasive biopsy surgery is not suitable, imaging examinations play a supplementary role in the diagnosis of calciphylaxis. Radiographic examination can reveal small-vessel calcification and reticular calcification patterns. Reticular calcification has the strongest association with calciphylaxis (odds ratio 9.4) and a specificity of nearly 90% (Shmidt et al., 2012). In a retrospective study, bone scintigraphy exhibited high sensitivity and specificity in the diagnosis of calciphylaxis. It also enabled the precise location and extent identification for microvessel calcification, as well as the evaluation of treatment response (Paul et al., 2017). A retrospective analysis of clinically and pathologically diagnosed calciphylaxis in CKD patients indicated that computed tomography (CT)-based radiomic models were more effective than plain radiography and bone scintigraphy (Yu et al., 2021).

### Multidimensional research system based on specific molecular makeup

The causal relationships between the harmful acute impacts, inducing ischemia and inflammatory responses, and rapidly progressive microvascular injuries in calciphylaxis patients are important. The multidimensional and artificial intelligence (AI)-technologies for multiple organs, with small samples and minimal amounts of tissue, have made it possible to conduct comprehensive analysis and tailor treatment plans for complex diseases (Wang et al., 2024b). Precision medicine attempts to select the optimal therapy for individual patients based on their specific molecular makeup. Technological advances in multiomics have increased the feasibility of quantifying molecular variability and identifying disease-specific deviations. This exploration paradigm will provide valuable knowledge about the mechanisms and will stimulate clinical translational research of calciphylaxis (Wang et al., 2024b).

## Pathogenesis of calciphylaxis

The current understanding of the pathogenesis of calciphylaxis is based on a large number of retrospective case reports, in which microvascular calcification, endothelial dysfunction, and microthrombosis are considered three critical processes (Rick et al., 2022b). Microvascular calcification is currently regarded as one of the main driving factors in the pathophysiological process (Colboc et al., 2019). At the same time, abnormalities in adipocyte proinflammatory cytokine signaling and endothelial injury also play important pathogenic roles (Weenig, 2008; Chen et al., 2014). Hypercoagulability is also implicated in the occurrence and development of calciphylaxis (Harris and Cropley, 2011; Dobry et al., 2018).

### Mechanisms of microvascular calcification

Microvascular calcification occurs as a consequence of the imbalance between factors that promote and inhibit calcification, ultimately leading to an inclination favoring calcification. It has been reported that the upregulation of bone morphogenetic protein 2 (BMP-2) and BMP-4 and the downregulation of carboxylated matrix Gla protein (c-MGP), inorganic pyrophosphate (PPi), and fetuin-A lead to matrix mineralization and the transdifferentiation of vascular smooth muscle cells (VSMCs) into an osteoblast-like phenotype (Nigwekar et al., 2018). In addition, polymorphisms in NT5E (rs4431401 and rs9444348) are overrepresented in calciphylaxis patients (Rothe et al., 2017).

MGP is an extracellular matrix protein synthesized by VSMCs and endothelial cells. c-MGP is a potent calcifiction inhibitor that inhibits the pro-calcification factors BMP-2 and BMP-4 (Schurgers et al., 2013). c-MGP deficiency leads to an upregulation of BMP-2 and BMP-4 expression in the skin, along with an enhancement of osteogenic transcription. This is accompanied by an elevation in the levels of runt-related transcription factor 2 (SP2) (Kramann et al., 2013; Nigwekar et al., 2017b). Carboxylation of MGP is dependent on vitamin K, and noncarboxylated MGP is unable to carry out its function of inhibiting calcification (Chen et al., 2017). Patients with CUA exhibit a reduced presence of circulating and skin-specific c-MGP (Kramann et al., 2013; Nigwekar et al., 2017a). Fetuin-A is an inhibitor of calcification. In CKD, particularly when combined with calciphylaxis, there is a notable accumulation of circulating calpain particles, which correlates with a significant deficiency of fetuin-A (Sowers and Hayden, 2010; Smith et al., 2013; Nigwekar et al., 2017b). Fetuin-A is involved in the formation of calpain particles that are transported in mineral nanocrystals and may be relevant to CUA (Schafer et al., 2003).

By being exposed to high phosphate levels, mature adipocytes calcify and induce calcification of VSMCs, probably through the release of vascular endothelial growth factor A (VEGF-A). VEGF-A is a potential adipokine that may trigger a procalcific response via BMP-4 (Hade et al., 2021). Mechanistic studies have demonstrated that galactoglucan and its sulfated derivative Sul-CDA-0.05 may target both BMPRIA and BMPRII, thereby blocking BMP/Smad/Id1 signaling and attenuating VEGF and its transcription factor (Huang et al., 2022). Inflammation is another key factor in the development of vascular calcification. The release of proinflammatory factors and oxidative stress leads to the formation of a procalcific environment (Voelkl et al., 2019) and activation of the RANK-mediated nuclear factor kappa-B (NF-κB) pathway (Zhou et al., 2023), resulting in vascular calcification (Kodumudi et al., 2020). In addition, imbalances in calcium and phosphorus metabolism, uremic toxins, and reactive oxygen species can promote the transformation of VSMCs to an active osteogenic phenotype (Sowers and Hayden, 2010).

### Mechanisms of endothelial injury and microthrombosis

Extensive intima–media calcification of small cutaneous arteries is associated with the disruption of the endothelial layer (Kramann et al., 2013). Vascular calcification promotes subintima–media fibrosis and thrombosis, while elevated levels of BMP promote endothelial cell proliferation and arterial stenosis (Zhou et al., 2023). After chronic dialysis, trauma, and chronic microvascular injury, inflammatory mediators such as tumor necrosis factor-alpha (TNF-α), interleukin 1 (IL-1), and IL-6 are elevated (Harris and Cropley, 2011; Yalin et al., 2013). These mediators contribute to endothelial dysfunction and produce a procoagulant effect by releasing tissue factor from the endothelium. As a result, there is a decrease in endothelial cell protein C and protein S receptor expression (Mehta et al., 1990), suppression of thrombomodulin levels (Zhou et al., 2023), and reduced production of natural vascular heparin-like molecules (Jeong and Dominguez, 2016). In addition, oxidative stress, lymphocyte activation, autoantibody production, and the release of proinflammatory factors can promote coagulation and decrease the expression of endothelial protein C and S receptors and thrombomodulin, leading to endothelial cell injury and dysfunction (Ding et al., 2020; Zhou et al., 2023). A hypercoagulable state plays an important role in the pathogenesis of CUA (Harris and Cropley, 2011; Dobry et al., 2018). Compared with normal subjects, patients with CUA have significantly lower levels of protein C, which may be associated with the hypercoagulable state and thrombosis in the presence of vascular injury (Mehta et al., 1990). Endothelial injury triggers platelet activation, as well as tissue factor-dependent thrombin and fibrin generation (Brouns et al., 2020), further promoting the hypercoagulable state. The severity of pain is usually disproportionate to the apparent skin damage. It has been reported that the formation of microvascular fibrin thrombi in skin biopsies of CUA correlates with the degree of skin pain (Dutta et al., 2019). 

## Animal models associated with calciphylaxis

Selye et al. (1961a) proposed the concept of ‘calciphylaxis’ for the first time. They documented the presence of trauma and calcium deposition in the skin and subcutaneous tissues of uremic rats after exposure to ‘sensitizing agents’ such as vitamin D and parathyroid hormone (Selye et al., 1961b). Furthermore, they observed that rats sensitized with dihydrotachysterol (DHT), a derivative of vitamin D, experienced a distinctive and often fatal musculocutaneous inflammation upon subsequent exposure to ferric dextran (Fe-Dex) alongside certain histamine liberators such as polymyxin (Selye et al., 1961a). However, calciphylaxis in humans is not a hypersensitivity reaction, but rather a cutaneous microvascular occlusive disorder caused by thrombosis and calcification (Nigwekar et al., 2015; Seethapathy et al., 2019). The rats in the experiments by Selye et al. (1961a) had cutaneous calcifications but not ischemic skin necrosis (Weenig, 2008; Jovanovich and Chonchol, 2016; Nigwekar et al., 2018). Glimcher et al. (1981) observed a significant increase in the levels of noncollagenous proteins during the induced pathological calcification of rat skin, using DHT and ovalbumin as stimuli for topical cutaneous calciphylaxis. Price et al. (2002) reported a calciphylaxis-associated model in Sprague Dawley rats. The model was created through the administration of subcutaneous injections of 400000 IU/kg body weight of vitamin D3 over a 3-day period, followed by local injection of 300 μg of FeCl_3_ (Price et al., 2002). Despite observing significant soft-tissue calcifications in the experimental animals, the researchers did not detect any small-artery or arteriolar calcifications (Nigwekar et al., 2015). There is currently no appropriate animal model that accurately replicates the clinical manifestations of calciphylaxis.

## Advances in the treatment of calciphylaxis

**Figure 3:**
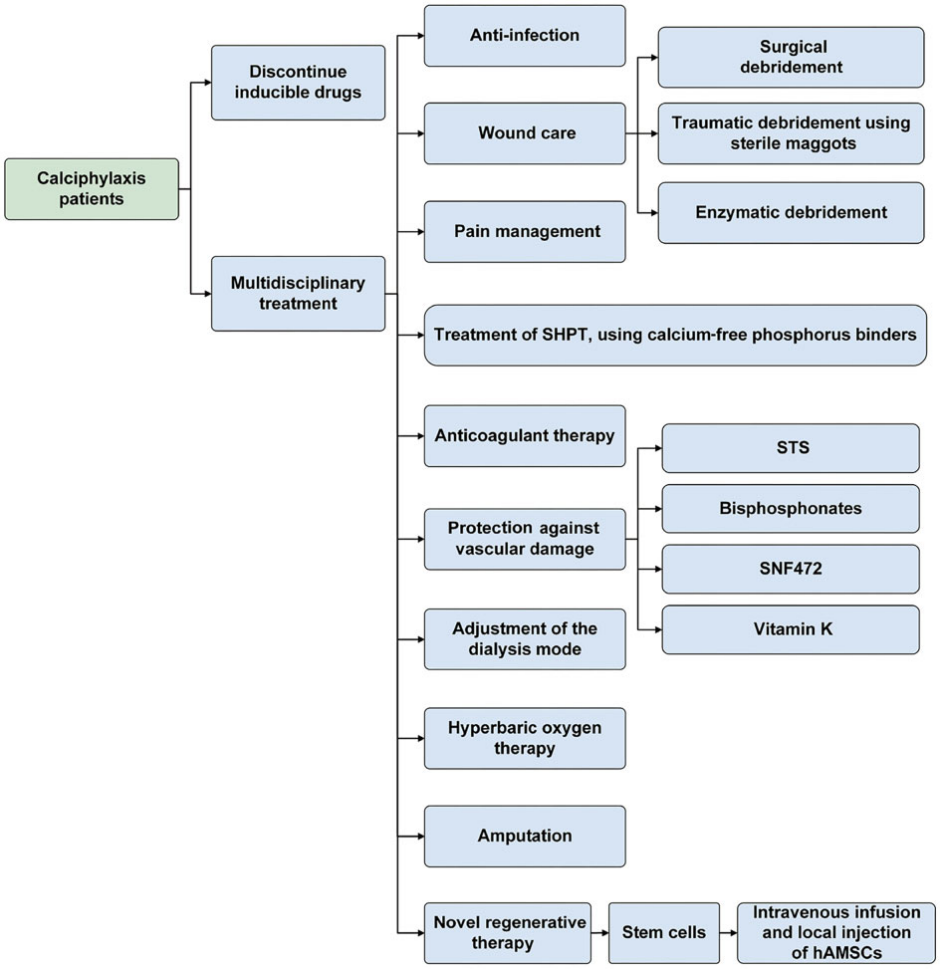
Current treatment options for calciphylaxis patients. STS, sodium thiosulfate; hAMSCs, human amnion-derived mesenchymal stem cells.

Figure 3 illustrates the current treatment options for patients with calciphylaxis.

### Wound care and symptomatic pain management

For calciphylaxis skin lesions, pain management and wound care are the first-line approaches to improve the condition (Chiriac et al., 2020). In most cases, even with the use of potent analgesics, achieving pain relief remains difficult. The primary goal of wound care is to remove exudate and necrotic tissue and prevent infection (Gallo Marin et al., 2023). Wound care methods include enzymatic debridement, traumatic debridement using sterile maggots, and surgical debridement (Vedvyas et al., 2012).

Considering the risk of koebnerization (Bretschneider et al., 2020), the appearance of new lesions in the traumatized skin area caused by tissue biopsy or surgery, local tissue ischemia leading to poor wound healing, and extreme pain at the lesion site, there is a debate about the use of surgical debridement as a wound management approach. A retrospective study showed no difference in 6-month survival rates among 101 patients with calciphylaxis, regardless of whether they underwent surgical debridement. However, the patients who did undergo surgical debridement showed a significant improvement in the overall survival throughout the entire follow-up period, particularly among those with CKD stage 5 or on dialysis (McCarthy et al., 2016). Another retrospective study involving 64 patients with calciphylaxis showed that the 1-year survival rate was 61.6% in the 17 patients who underwent surgical debridement, compared with 27.4% in the 46 patients who did not undergo surgical debridement (*P* = 0.008) (Weenig et al., 2007).

### Pharmacotherapy

Pharmacotherapy for calciphylaxis is summarized in Table 2.

**Table 2:** Pharmacotherapy options available for the treatment of calciphylaxis.

Treatment	Mechanism	Clinical trials				Evaluation
		Regimen	Study title (identifier)	Status	Locations	
STS	STS chelates calcium salts and reduces calcification in adipocytes and VSMCs	STS (25 g) intravenously administered each HD session (three times weekly) for 3 weeks	A Phase 3 clinical trial of intravenous STS in acute calciphylaxis patients (CALISTA) (NCT03150420)	Phase 3, terminated	Mayo Clinic, USA, and 20 more study locations	STS can cure or alleviate clinical symptoms in patients with CUA. It was safe, effective, and well tolerated (Cicone et al., 2004; Nigwekar et al., 2013; Strazzula et al., 2013; Zitt et al., 2013; Lu et al., 2022).
						Two systematic reviews and meta-analyses found no association between the improvement of skin lesions and the survival benefits of STS treatment for calciphylaxis (Udomkarnjananun et al., 2019; Wen et al., 2023).
SNF472	SNF472 binds to HAP and inhibits further HAP crystallization, as well as directly inhibits calcium phosphate crystal formation and aggregation	SNF472 (7 mg/kg intravenously during HD) three times a week for 24 weeks	Phase 3 study of SNF472 for calciphylaxis (NCT04195906) Phase 2 study with SNF472 in calciphylaxis patients (NCT02790073)	Phase 3, completed Phase 2, completed	AKDHC Medical Research Services, USA, and 57 more study locations Fresenius Medical Services, USA, and two more study locations	SNF472 was well tolerated and can promote wound healing and improve pain and quality of life in patients with calciphylaxis (Perelló et al., 2020; Sinha et al., 2022). However, the improvements in BWAT-CUA and visual analogue scale are comparable between SNF472 and placebo (Sinha et al., 2024).
Vitamin K	Vitamin K can increase the circulating levels of c-MGP, which is a potent inhibitor of vascular calcification	Vitamin K1 (phytonadione) 10 mg orally three times a week after dialysis for 12 weeks	Evaluation of vitamin K supplementation for calcific uremic arteriolopathy (NCT02278692)	Completed	Massachusetts General Hospital, USA	Vitamin K deficiency was associated with vascular calcification, and vitamin K supplementation can promote symptomatic relief and improve disease status (Christiadi and Singer, 2018; Mizuiri et al., 2019).
Lanthanum carbonate	Lanthanum carbonate is a nonaluminum, noncalcium phosphate binder and reduces serum phosphate levels, which are the proposed etiologic mechanism of injury and vascular calcification in patients with CUA	Lanthanum carbonate administered orally in a dose of 1500–3750 mg daily, divided into doses with meals, for 12 weeks	Efficacy of lanthanum carbonate in calciphylaxis (NCT01289626)	Phase 1, completed	University of Wisconsin Hospitals, USA	Lanthanum carbonate appears to be effective as an adjunctive therapy to improve calciphylaxis lesions and symptom burden (Chan et al., 2014).
Rheopheresis	This double-filtration plasmapheresis eliminates a defined spectrum of high-molecular-weight proteins from human plasma, including relevant factors for vascular inflammation and thrombose	Induction treatment: three apheresis sessions during the first week and then two apheresis sessions each week for 3 weeks; maintenance treatment: one apheresis session per week until the 11th week	Rheopheresis as adjuvant treatment of calciphylaxis (RHEO-CAL) (NCT04654000)	Enrolling by invitation	University Hospital, Lille, France	Rheopheresis was reported in a case to be used as an adjuvant treatment for severe calciphylaxis to reduce cutaneous scarring and limit infection-associated disease (Bouderlique et al., 2018).
hAMSCs	Inhibition of vascular calcification, wound repair, immunomodulation, and hypercoagulability improvement	Intravenous infusion combined with local injection to skin lesions	Stem cells for uremic calciphylaxis patients (NCT04592640)	Active, recruiting	The First Affiliated Hospital with Nanjing Medical University, Jiangsu Province Hospital, China	hAMSCs have been innovatively utilized to rescue CUA patients who are unresponsive to traditional therapies. They were well tolerated and effective, promoting wound healing, improving clinical symptoms, and regulating immunity (Qin et al., 2022; Wang et al., 2022a; Bian et al., 2023; Lu et al., 2025).
Bisphosphonates	Bisphosphonates are analogues of PPi, which is a potent inhibitor of calcium crystallization and prevents HAP from forming *in vitro*, and serve as an endogenous inhibitor of calcification	It is recommended to administer intravenously during HD	/	/	Hospital Clinic of Barcelona, Spain	Bisphosphonate treatment improves pain and skin lesions in patients with CUA and is well tolerated (Torregrosa et al., 2012, 2015). A systematic review and meta-analysis found no significant mortality benefit of bisphosphonates treatment for CUA (Udomkarnjananun et al., 2019).
LMWH	Anticoagulation	LMWH 250 U/kg, administered twice daily (Carter et al., 2016)	/	/	New York Medical College, USA	A CUA patient experienced a significant improvement in symptoms, and the wounds on the lower limbs completely healed after adding a 5-month course of treatment with LMWH (Carter et al., 2016).
tPA	Dissolution of blood clots	Low-dose tPA protocol (Sewell et al., 2004): tPA 10 mg/day for 14 days, administered via slow intravenous infusion, i.e. 10 mg tPA reconstituted in 200 ml of isotonic NaCl solution for infuse over 4 h (2.5 mg/50 ml per hour)	/	/	Mayo Clinic, USA	In a retrospective analysis of 63 calciphylaxis patients, the survival rate of those who received tPA treatment was found to be 30% higher than that of the control group, although this difference was not statistically significant (El-Azhary et al., 2013).

HD, hemodialysis; LMWH, low-molecular-weight heparin; tPA, tissue plasminogen activator; hAMSCs, human amnion-derived mesenchymal stem cells.

#### Inhibition of vascular calcification

(I) Sodium thiosulfate. In 2004, the use of intravenous sodium thiosulfate (STS) as a systemic treatment for calciphylaxis was initially reported (Cicone et al., 2004). STS possesses the capability to chelate calcium salts, thereby reducing calcification in adipocytes and VSMCs, which in turn improves skin lesions (Strazzula et al., 2013; Zitt et al., 2013; Peng et al., 2018; Seethapathy and Noureddine, 2019). The intravenous administration of STS has the potential to reduce reactive oxygen species while promoting vasodilation (Vedvyas et al., 2012). The dosage of STS varies in different studies; however, the classic regimen typically involves adding 25 g of STS to a 100-ml solution, which is then administered intravenously three times a week during the final 30–60 min of hemodialysis (Nigwekar et al., 2018). A study involving 27 CUA patients treated with STS demonstrated that complete resolution of the condition occurred in 52% of the patients, while 19% of the patients experienced partial improvement (Zitt et al., 2013). Another study involving 172 CUA patients who had received intravenous STS treatment reported a median dose of 25 g. Complete resolution of CUA occurred in 26.4% of the patients, while a significant improvement was observed in 18.9% of the patients (Nigwekar et al., 2013). A case report outlined the successful treatment of CUA with intravenous STS at a low dose range of 3.2–6.4 g per infusion over a 6-month period (Lu et al., 2022). In addition, intravenous STS has been reported to be effective in peritoneal dialysis patients and in calciphylaxis patients with normal renal function (Almafragi et al., 2009; Lu et al., 2022; Zhou et al., 2023). However, STS is ineffective in some CUA patients (Nigwekar et al., 2015), and its long-term usage may elevate the risk of fractures (Dobry et al., 2017). A recent meta-analysis has found no significant association between the administration of intravenous STS and improvements in skin lesions or survival benefits in CUA patients (Wen et al., 2023). There have been some case reports that used STS to successfully treat NUC (Ning et al., 2013). According to Nigwekar et al. (2015), STS can successfully be used for the treatment of NUC, with intravenous STS, 12.5 or 25 g, administered 4–5 days a week in isolation or in combination with weekly intralesional STS (Nigwekar et al., 2015). Intralesional STS is also a potential alternative treatment for calciphylaxis, with low risk and highly targeted use in the skin (Granja et al., 2024). However, a consensus regarding the optimal treatment duration has not yet been achieved.

(II) Bisphosphonates are analogs of pyrophosphate. They have the ability to selectively bind to HAP in bone, resulting in the inhibition of osteoclast activity and a reduction in bone resorption. Not only do bisphosphonates possess anti-inflammatory effects, but they can also alleviate skin lesions and reduce pain in individuals afflicted with calciphylaxis (Torregrosa et al., 2015; Harris et al., 2018). In a prospective case series study, the administration of bisphosphonates for 2–4 weeks effectively arrested the advancement of skin lesions in calciphylaxis. Furthermore, enhancements in pain relief and wound healing were observed at an accelerated rate (Torregrosa et al., 2012). Another prospective study involving 11 patients with calciphylaxis showed that the inclusion of bisphosphonates after 2–4 weeks of treatment decelerated the progression of skin lesions in all patients. This correlated with significant enhancements compared with patients undergoing solely supportive therapy (i.e. debridement, low-calcium dialysate) (Torregrosa et al., 2015). Bisphosphonates offer a viable treatment option for calciphylaxis patients (Torregrosa et al., 2012; Perelló et al., 2020; Sinha et al., 2022; Gallo Marin et al., 2023). As these drugs are primarily excreted via the kidneys, in patients with CKD, nephrotoxicity is associated with the rate and dose of drug infusion. Therefore, it is crucial to adjust the infusion duration. For CKD stages 4–5 patients, the recommended dosage is halved, and the infusion rate is reduced. For patients on hemodialysis, it is recommended to administer during hemodialysis (Torregrosa and Ramos, 2010). Both STS and bisphosphonates lack prospective randomized clinical trials, and thus there is no significant evidence of a mortality benefit relative to other commonly utilized treatments (Udomkarnjananun et al., 2019).

(III) SNF472 is the hexasodium salt of inositol hexaphosphate, which serves as a novel inhibitor of HAP crystals. It can reduce the formation and deposition of ectopic calcium, delaying coronary and aortic valve calcification (Perelló et al., 2020; Sinha et al., 2021, 2022). Intravenous administration of SNF472 effectively inhibits the progression of cardiovascular calcification in uremic rats by up to 80% (Ferrer et al., 2018). SNF472 can inhibit vascular calcification, and its efficacy and safety have been confirmed in subgroup analyses of the CALIPSO trial (Raggi et al., 2020; Yang et al., 2024). In an open-label study, SNF472 improved pain, wound healing, and quality of life among calciphylaxis patients over a 12-week period (Perelló et al., 2020). The phase III clinical trial, CALCIPHYX, is currently in progress, providing evidence for the promising clinical potential of SNF472, a novel inhibitor of vascular calcification named hexasodium fytate (Sinha et al., 2022). However, recent research suggests that the improvements in BWAT-CUA and visual analogue scale are comparable between SNF472 and placebo (Sinha et al., 2024).

(IV) Vitamin K. ESKD patients receiving hemodialysis often experience a relative deficiency of vitamin K (Mizuiri et al., 2019). There have been reports of complete recovery in patients with calciphylaxis and vitamin K deficiency following vitamin K supplementation and increased frequency of hemodialysis (Christiadi and Singer, 2018). However, excessive supplementation of vitamin K may lead to an elevated risk of coagulation. It is imperative to develop personalized treatment plans.

*Anticoagulation.*  Carter et al. (2016) reported a case of calciphylaxis that did not respond to conventional medications (cinacalcet, sevelamer, and STS) and surgical debridement of skin wounds. Laboratory tests revealed the presence of lupus anticoagulant and anticardiolipin antibodies, along with cryoglobulinemia. After a 5-month course of treatment with low-molecular-weight heparin (LMWH, 250 U/kg, administered twice daily), the patient experienced a notable improvement in symptoms, and the wounds on the lower limbs completely healed. Furthermore, tissue plasminogen activator (tPA) has shown promise as a potential adjunctive therapy for calciphylaxis. In a retrospective analysis of 63 calciphylaxis patients, the survival rate of those who received tPA treatment was 30% higher than that of the control group. However, this difference was not statistically significant (El-Azhary et al., 2013). In a case of NUC associated with cholangiocarcinoma, despite receiving treatment with LMWH (130 U/kg, twice daily) and vitamin K, the patient succumbed to the condition (Riegert-Johnson et al., 2001).

#### Other treatment

Discontinuation of triggering medications, anti-infection treatment, and enhanced nutritional support are important treatment approaches for calciphylaxis (Nigwekar et al., 2018; Rick et al., 2022a; Gallo Marin et al., 2023). Increasing dialysis adequacy has been reported to show potential therapeutic effects in a 51-year-old female CUA patient. By increasing the dialysis frequency to six times per week and combining it with wound care and STS, extensive ulcers completely healed (Adapa et al., 2020). Hyperbaric oxygen therapy may be beneficial in wound healing and reducing mortality rates in the treatment of calciphylaxis (Biglione et al., 2024). In addition, clinical cases have demonstrated the use of human amnion-derived MSCs (hAMSCs) to improve clinical symptoms and promote wound healing in patients with calciphylaxis (Qin et al., 2022).

In summary, we provide a comprehensive overview of the literature reviews outlining the disparities between CUA and NUC in Table 3.

**Table 3:** A comprehensive overview of the literature reviews outlining the disparities between CUA and NUC.

Author (year)	Study	Participants (*n*)	Mean age/range (years)	Female (%)	Mortality rate (%)	Conclusion
**CUA**						
Hayashi et al. (2012)	Case–control study	28	58.4	57.1	/	Warfarin therapy and lower serum albumin levels are strong risk factors for CUA patients in Japan.
Nigwekar et al. (2016)	Case–control study	1030	54	67	27% at 6 months, 45% at 12 months	CUA has a predilection for white, female, diabetic, and obese patients. CUA triggers such as VKA or vitamin K deficiency, skin trauma, and mineral bone abnormalities should be avoided.
McCarthy et al. (2016)	Retrospective study	101	60	80.2	13.4%, 52%, 62.9%, and 76.9% at 6, 12, 36, and 60 months, respectively	Surgical debridement and subtotal parathyroidectomy in stage 5/5D CKD patients with hyperparathyroidism and calciphylaxis were significantly effective treatments. Treatments with tPA, STS, and hyperbaric oxygen therapy were not associated with higher mortality.
Chen et al. (2017)	Retrospective cohort study	57	60	79	/	Dermal angioplasia, a potential marker of chronic low-grade ischemia, was a frequent microscopic finding in calciphylaxis. Histopathologic changes in patients with and without CKD were indistinguishable to imply a final common pathogenic pathway in both uremic and non-uremic calciphylaxis.
Renner et al. (2017)	Retrospective study	699	65–80	37	/	Most calciphylaxis patients with a male:female ratio of 1.7:1 were at an age range of 65–80 years. They showed ESKD with need of dialysis and presented with the resulting complications.
Harris et al. (2018)	Retrospective study	24	60.5	71	41% at 12 months, 64% at 30.5 months	A multi-intervention approach, intensive HD, STS, wound care, analgesics, and discontinuation of trigger medications can be successful in treating patients with severe CUA lesions.
Dobry et al. (2018)	Case–control study	38	>18	58	/	Presence of lupus anticoagulants and combined thrombophilias are risk factors for the development of calciphylaxis in ESKD patients
Dutta et al. (2019)	Retrospective study	70	58	60	/	In calciphylaxis, ulceration, induration, and erythema were common cutaneous clinical features, while cutaneous microvascular calcification and necrosis were common pathological features. The presence of fibrin thrombi in skin biopsies was associated with pain severity. The stage of a skin lesion positively correlated with the presence of necrosis on histological analyses.
Russ et al. (2019)	Retrospective study	15	64.8	60	73.3% at 12 months	Treatment with VKA and liver dysfunction are most important concomitant factors in development of calciphylaxis. Early use of DOACs instead of VKA might be beneficial and reduce the incidence of calciphylaxis.
Colboc et al. (2019)	Retrospective, multicenter cohort study	36	64	72	33%	Calcifications of CUA are composed of pure calcium phosphate apatite, located circumferentially, mostly in the intima of normal-appearing vessels, and often associated with interstitial deposits, which is different from arteriolosclerosis calcifications, suggesting different pathogenetic mechanisms.
Liu et al. (2022b)	Cross-sectional survey	58	53.85	31.25	/	The prevalence of calciphylaxis in Chinese HD patients was 1.24% according to regional epidemiological survey.
Qin et al. (2022)	Original research	1	34	Female	Passed away after 20 months	Intravenous and local intramuscular injections of hAMSCs, as well as external application of hAMSC supernatant, could be a novel candidate for regenerative treatment in CUA patients. The effects due to inhibiting vascular calcification, stimulating angiogenesis and myogenesis, anti-inflammatory and immune modulation, multidifferentiation, re-epithelialization, and restoration of integrity.
Bian et al. (2023)	Case report	1	34	Female	Passed away after 20 months	Hypercoagulability of CUA can be effectively improved by hAMSC treatment.
Liang et al. (2023)	Case report	1	72	Female	Passed away after 1 month	DOACs are now the first alternatives to VKA in ESKD patients with atrial fibrillation. The early use of DOACs may be beneficial in reducing the incidence of calciphylaxis. Selecting biological valves that need only 2–3 months of warfarin treatment might be essential for ESKD patients.
**NUC**						
Yu et al. (2017)	Case series	3	40–80	66.7	All three cases survived	Lesions of warfarin-associated calciphylaxis were usually located below the knees. Calcifications were most often noted in the tunica media or in the vessel lumen and tunica intima. The survival rate on hospital discharge was remarkably high.
Altman and Shinohara (2019)	Retrospective study	16	22–87	94	25% at 12 months	Sex and anticoagulant use are two important risk factors for NUC. STS therapy is possibly beneficial to improve survival rate for NUC.
Truong et al. (2019)	Case report	1	75	Female	Survival during follow-up	Pamidronate infusions can be an effective treatment option for NUC.
Shahzad et al. (2021)	Case report	1	65	Female	Passed away after 4 months	Calciphylaxis can occur following acute kidney injury. Multiple risk factors for NUC include obesity, female gender, hyperparathyroidism, elevated calcium–phosphorous product, hypoalbuminemia, autoimmune diseases, diabetes, warfarin, steroids, and chemotherapy medications.
Mathur et al. (2021)	Case report	1	34	Female	Survival during follow-up	STS is considered as first-line therapy for COVID-19 patients with calciphylaxis.
Ababneh et al. (2022)	Retrospective study	35	67	86	/	NUC has a predilection for obese postmenopausal females. Undiagnosed hyperparathyroidism has a possible association with NUC. Lupus anticoagulant was positive in most patients. NUC biopsies are more likely to display extravascular calcium deposition.
Wiltfang et al. (2022)	Case report	1	69	Male	Survival during follow-up	Combining STS and iloprost showed successful improvement in NUC.

HD, hemodialysis.

## Opportunities for MSCs in the treatment of calciphylaxis

### Definition, sources, and clinical applications of MSCs

MSCs represent a specific category of multipotent stem cells that can be sourced from various human tissues and organs. Initially identified in mouse bone marrow (Friedenstein et al., 1976), these cells were later named MSCs (Caplan, 1991). MSCs can be derived from nearly all tissues, including human amnion, adipose tissue, peripheral blood, endometrial polyps, bone marrow, neonatal placenta, and umbilical cord tissues, etc. (Xu et al., 2024). MSCs are characterized by their tri-lineage differentiation potential (osteogenic, adipogenic, and chondrogenic), cell surface expression of CD90, CD105, and CD73, and the absence of cell surface markers CD45, CD34, CD14, CD79, and human leukocyte antigen-DR (HLA-DR) (Ankrum et al., 2014). These cells exhibit the unique capability of self-renewal and the potential to differentiate into various cell lineages (Salem and Thiemermann, 2010). MSCs migrate to the site of tissue injury through their homing ability and play important roles in processes such as cell proliferation, differentiation, immune regulation, angiogenesis, wound healing, tissue regeneration, and inhibition of vascular calcification (Xie et al., 2019; Raghuram et al., 2020; Zhang et al., 2021; Wang et al., 2022b). They do so by secreting immunomodulatory factors, cytokines, growth factors, extracellular vesicles, and other bioactive substances (Fu et al., 2019; Xunian and Kalluri, 2020). They have potential therapeutic prospects in various diseases such as COVID-19, cardiovascular diseases, skin injuries, and ischemic diseases (Zhou et al., 2020; Gabel et al., 2021a), and they are widely used as seed cells in tissue engineering and regenerative medicine (Mahla, 2016). Here, we analyze the application prospects of MSCs in the treatment of calciphylaxis.

### Potential mechanisms of MSCs for the treatment of calciphylaxis

**Figure 4:**
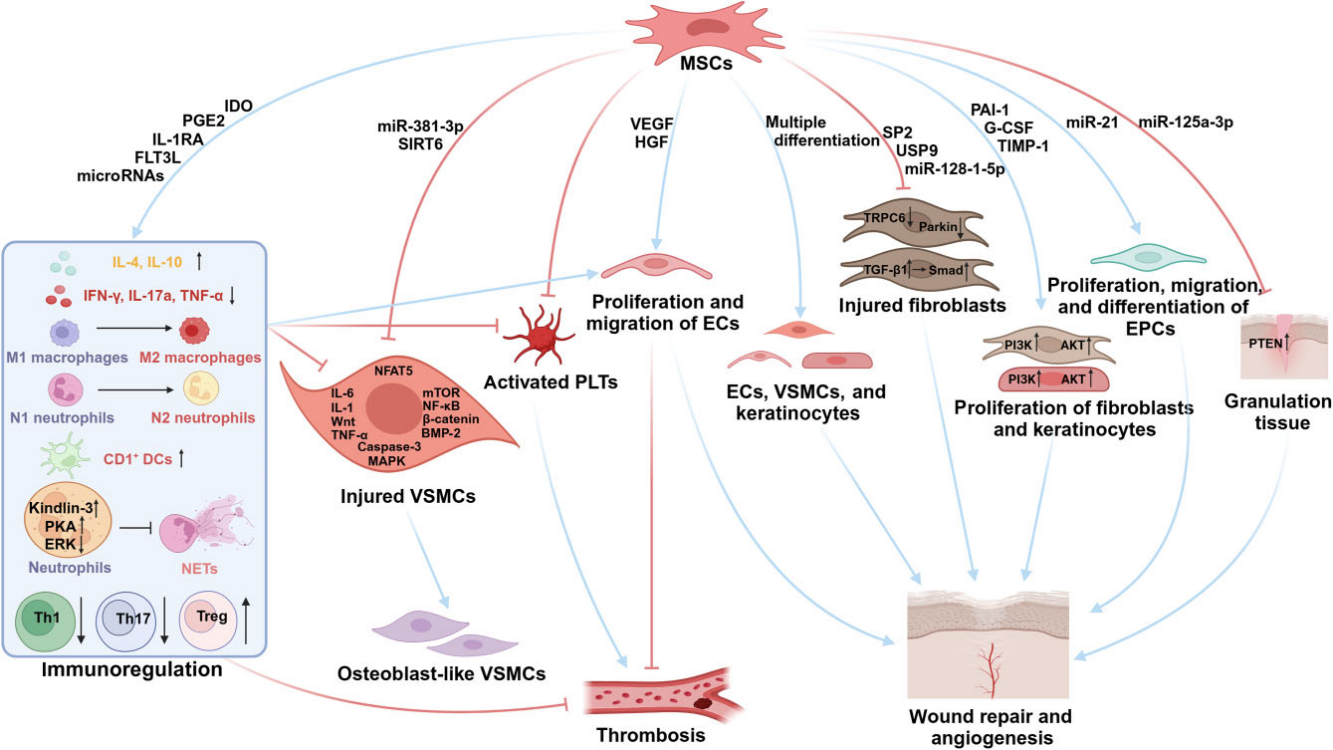
Potential mechanisms underlying MSC therapy for calciphylaxis. MSCs can directly affect primary and adaptive immune cells, promoting differentiation toward anti-inflammatory phenotypes, inhibiting the release of proinflammatory factors while enhancing the release of anti-inflammatory factors. This immunomodulatory function helps inhibit vascular calcification, reduce inflammation and thrombosis, and promote neovascularization. MSCs and their exosomes inhibit vascular calcification by suppressing signaling pathways, such as mTOR, MAPK, and Wnt, inhibiting apoptosis and inflammation through the secretion of cytokines, and altering microRNA profiles in VSMCs. MSCs also inhibit the activation of platelets and PETN expression in granulation tissue. With multidirectional differentiation potential, MSCs play an important role in wound repair by differentiating into endothelial cells, VSMCs, and fibroblasts. Furthermore, MSCs influence the proliferation and migration of keratinocytes, fibroblasts, etc., further promoting skin wound repair. PLTs, platelets; ECs, endothelial cells.

The potential mechanisms underlying MSC therapy for calciphylaxis are illustrated in Figure 4.

#### Immunomodulation

MSCs can directly influence innate and adaptive immune cells, such as macrophages, natural killer cells, dendritic cells (DCs), mast cells, T lymphocytes, and B lymphocytes. They do so by modulating and restoring immune homeostasis through the blocking of the cascade activity of immune reactions (Wang et al., 2022b). hAMSCs can exert an immunomodulatory effect through the secretion of cytokines such as indoleamine 2,3-dioxygenase (IDO) and prostaglandin E2 (PGE2), as well as cell-to-cell contact, inhibiting the proliferation of stimulated lymphocytes and T cells (Bulati et al., 2020). In addition, previous studies have found that MSCs and their derived exosomes can reduce inflammation by converting macrophages from a proinflammatory M1 phenotype to an anti-inflammatory M2 phenotype via microRNAs such as miR-125b-5p, miR-130b-3p, miR-27a-3p, and miR-24 (Bulati et al., 2020; Wang et al., 2020b; Hou et al., 2024). Intravenous injection of human umbilical cord MSC exosomes (HUMSC-Exo) in a mouse model of autoimmune encephalomyelitis showed a significant decrease in proinflammatory cytokine levels such as IL-17a, TNF-α, and interferon-γ (IFN-γ), and an increase in anti-inflammatory factors such as IL-4 and IL-10 (Ahmadvand Koohsari et al., 2021). Intraperitoneal injection of HUMSCs can induce neutrophil polarization toward the N2 phenotype, balancing the expression of proinflammatory and anti-inflammatory factors and alleviating murine inflammatory bowel disease (Wang et al., 2020a). A study on a rat model for treating spinal cord injury has shown that MSC-Exo can significantly reduce the infiltration of neutrophils and the formation of neutrophil extracellular traps (NETs) by delivering miR-125a-3p, thereby alleviating the spinal cord injury (Morishima et al., 2024). In a mouse model of serum-induced metastatic arthritis, it has been demonstrated that human gingiva-derived MSCs inhibit neutrophil infiltration and NET formation, thereby attenuating inflammation via the PGE2–protein kinase A (PKA)–extracellular signal-regulated kinase (ERK) axis (Zhao et al., 2024).

A clinical study on the treatment of systemic lupus erythematosus has shown that MSCs can promote the proliferation of tolerogenic CD1c^+^ DCs and inhibit their apoptosis by upregulating FMS-like tyrosine kinase 3 ligand (FLT3L), thereby reducing inflammation levels in patients (Yuan et al., 2019). Single-cell RNA sequencing demonstrated that immunosuppressive CD11b^mid^Ly6C^mid^Ly6G^lo^ cells, differentiated from bone marrow MSC-induced bone marrow cells, comprised seven cellular subtypes. Among these, CSF-1R^hi^CD11b^mid^Ly6C^mid^ cells inhibited Th1- and Th17-cell differentiation and promoted CD4^+^CD25^+^Foxp3^+^ Treg differentiation in both culture and a mouse model of experimental autoimmune uveoretinitis (Lee et al., 2024).

#### Inhibition of vascular calcification

Bone marrow MSC exosomes (BMSC-Exo) inhibit high phosphorus-induced VSMC calcification *in vitro* by modifying microRNA profiles and affecting the mammalian target of rapamycin (mTOR), mitogen-activated protein kinase (MAPK), and Wnt signaling pathways (Guo et al., 2019). Increased serum levels of high mobility group box 1 (HMGB1) promote vascular calcification through the Wnt/β-catenin pathway (Jin et al., 2018). BMSC-Exo can reduce HMGB1 levels through the sirtuin 6 (SIRT6)–HMGB1 deacetylation pathway, inhibiting high phosphate-induced arterial calcification and improving renal function (Wei et al., 2021). Liu et al. (2022a) found that BMSC-derived exosomal microRNA (miR-381-3p) inhibited human aortic smooth muscle cell calcification stimulated by high phosphate and vascular calcification in a 5/6 nephrectomy rat model by suppressing the expression nuclear factor of activated T cells 5 (NFAT5). BMSC-Exo also inhibited high phosphate-induced VSMC calcification by regulating the NF-κB axis (Liu et al., 2021).

MSC-conditioned medium (MSC-CM) has been found to inhibit VSMC calcification by blocking the BMP-2–Smad1/5/8 signaling pathway (Wang et al., 2018a) and reducing the expression of TNF-α, IL-1, and IL-6, as well as caspase-3, in VSMCs (Wang et al., 2018b). Intravenous injection of mouse adipose-derived MSCs (ADMSCs) may inhibit thoracic aortic calcification in rats with high adenine-induced CKD by improving renal function and reducing blood phosphate levels (Yokote et al., 2017).

#### Improvement of hypercoagulability

*In vitro* studies have shown that human ADMSC microspheres can improve the progression of atherosclerosis by inhibiting oxidative stress damage, cell apoptosis, endothelial dysfunction, inflammation, and lipid accumulation (Yang et al., 2023). Stimulation of platelets with human placenta-derived MSCs (HPMSCs) or their secretome has been found to reduce oxidized low-density lipoprotein-induced CD36-mediated platelet activation, inhibit thrombus formation, and exert protective effects in atherosclerosis (Al Subayyil et al., 2021).

An *in vivo* study in mice has demonstrated that MSCs inhibit NET release and reduce venous thrombus formation by upregulating kindlin-3 signaling (Zhu et al., 2021). Intravenous injection of human UMSCs in MRL/lpr mice showed reduced levels of plasma inflammatory cytokines and thrombosis markers, improving the tendency of inflammatory thrombosis formation in mice (Cai et al., 2015). In a rat model of lipopolysaccharide-induced disseminated intravascular coagulation, pretreatment with BMSCs reduced coagulation and alleviated organ dysfunction by improving the peripheral immune response (Wang et al., 2017).

#### Wound repair

MSCs are involved in the entire process of wound healing, promoting angiogenesis, tissue repair, and closure of chronic nonhealing wounds (Raghuram et al., 2020; Gao et al., 2022). MSCs can promote neovascularization through the secretion of cytokines, such as VEGF and hepatocyte growth factor (HGF), and multiple differentiation into keratinocytes, endothelial cells, or VSMCs while simultaneously forming perivascular cells (Zhao et al., 2017; Ertl et al., 2018). The use of HUMSC-derived small extracellular vesicle (HUMSC-sEV) has been shown to promote skin wound healing in diabetic rats. The mechanism involves delivering SP2 and the deubiquitinating enzyme ubiquitin-specific peptidase 9 (USP9) to enhance the expression of transient receptor potential cation channel-6 (TRPC6) and Parkin in dermal fibroblasts. This restores calcium ion influx and downstream signaling pathways, promotes mitochondrial autophagy, and aids in the recovery of mitochondrial function (Wang et al., 2024a). Skin-derived ATP-binding cassette subfamily B member 5^+^ (ABCB5^+^) MSCs attenuate the inflammatory response and accelerate angiogenesis by secreting an IL-1 receptor antagonist (IL-1RA). This antagonist converts M1 macrophages to an M2 phenotype in a chronic iron-loaded mouse model, mimicking the human chronic venous ulcer wound model (Vander Beken et al., 2019). An intravenous injection of human ADMSC-CM accelerates wound closure, improves re-epithelialization, and increases blood vessels in the wound bed in the type 2 diabetic foot ulcer model in mice (De Gregorio et al., 2020). Subcutaneous injection of hAMSCs and hAMSC-CM can accelerate re-epithelialization of thermal burn wounds and promotes wound healing by activating the phosphatidylinositol 3-kinase/V-akt murine thymoma viral oncogene homolog (PI3K/AKT) signaling pathway through the delivery of cytokines such as plasminogen activator inhibitor 1 (PAI-1), granulocyte colony-stimulating factor (G-CSF), and tissue inhibitor of metalloproteinases 1 (TIMP-1) (Li et al., 2019). In a rat model of a cranial defect, HUMSC-Exo were found to promote angiogenesis through proliferation, migration, and differentiation of endothelial progenitor cells (EPCs) via miR-21 (Zhang et al., 2021). In a C57BL/6 mouse model of a full-thickness skin defect, exosomal miRNA-125a-3p from human ADMSCs inhibited phosphatase and tensin homolog (PTEN) gene expression in the granulation tissue of the wound, thereby promoting angiogenesis and wound healing (Pi et al., 2022). Furthermore, MSC-Exo can facilitate the healing of diabetic ulcers by modulating the TGF-β1/Smad signaling pathway in fibroblasts through miR-128-1-5p and by promoting the polarization of macrophages from the M1 to the M2 phenotype through miR-146a-5p (Liang et al., 2024; Zhou et al., 2024).

A follow-up study of 14 patients with diabetic foot ulcers and peripheral arterial disease showed that local and intravenous administration of HUMSCs shortened wound healing time and prevented amputation events within 3 years (Zhang et al., 2022). Muscle injection of HPMSCs in patients with severe limb ischemia resulted in a significant decrease in resting pain scores and a significant increase in pain-free walking distance after 24 weeks of treatment. In addition, one patient showed increased collateral vessel formation (Wang et al., 2021). Recently, a randomized controlled study has reported that local application of ABCB5^+^ MSCs intramuscularly in patients with a refractory diabetic foot ulcer promoted lesion neovascularization and accelerated wound healing, with no treatment-related adverse events observed. The treatment worked through paracrine VEGF and transdifferentiation into endothelial lineage cells (Kerstan et al., 2022).

## hAMSCs as a potential treatment for calciphylaxis patients

### Definition and sources of hAMSCs

hAMSCs are a type of MSC isolated from the amniotic membrane, the innermost layer of the placenta, which is in direct contact with the amniotic fluid and fetus (Lindenmair et al., 2012; Li et al., 2020). The amniotic membrane consists of an epithelial layer and a mesenchymal stromal layer, with hAMSCs residing in the latter.

### Comparative analysis of hAMSCs with other MSCs

#### Origin and ethical considerations

While BMSCs require invasive bone marrow aspiration and ADMSCs necessitate liposuction, hAMSCs are derived from medical waste (placenta), thereby circumventing ethical and procedural challenges (Yusoff et al., 2015; Li et al., 2020). In addition, parturients are typically young women, and thus, the age-related heterogeneity of hAMSCs may be relatively lower than that of stem cells from other sources (Li et al., 2020).

#### Proliferation, differentiation capacity, and paracrine activity

hAMSCs exhibit superior proliferation rates and longevity in culture compared to adult-derived MSCs. For instance, dental pulp stem cells display higher passage numbers but have limited differentiation lineages compared to hAMSCs (Yusoff et al., 2015). Umbilical cord-derived MSCs (UC-MSCs) share this advantage of circumventing ethical and procedural challenges, but exhibit different differentiation potentials; hAMSCs demonstrate a stronger osteogenic capacity compared to UC-MSCs, although this capacity is less than that of BMSCs (Wu et al., 2018; Li et al., 2020). hAMSCs, insulin-like growth factor-1 (IGF-1), and tumor necrosis factor-stimulated gene-6 (TSG-6) enhance anti-inflammatory responses, promote angiogenesis, reduce fibrosis, and support tissue regeneration (Li et al., 2020; Ragni et al., 2021; Zuliani et al., 2021). hAMSC-CM has demonstrated efficacy in mitigating asthma-related inflammation and fibrosis, outperforming ADMSC-CM in the production of anti-inflammatory cytokines (Dalouchi et al., 2021; Ragni et al., 2021).

#### Immunogenicity and tumorigenicity

The human amniotic membrane emerges during embryonic development (Days 7–8) as a bilayered structure formed through interactions between the extraembryonic mesoderm and trophoblast cells. This embryonic origin confers unique low immunogenicity and immunomodulatory properties to hAMSCs (Gu et al., 2013). The hAMSCs lack the expression of human major histocompatibility complex antigens, such as HLA class I antigens, which contributes to their low immunogenicity (Bailo et al., 2004; Evangelista et al., 2008). Additionally, hAMSCs exhibit low tumorigenicity due to the absence of telomerase expression (Miki et al., 2005; Parolini et al., 2009; Meng et al., 2019). These properties make hAMSCs suitable for allotransplantation to facilitate tissue regeneration.

### Preliminary preclinical and clinical applications of hAMSCs in the treatment of calciphylaxis

We have innovatively utilized hAMSCs to rescue CUA patients who were unresponsive to traditional therapies, resulting in favorable therapeutic outcomes (Wang et al., 2022a; Pan et al., 2023). Prior to treatment, we conducted quality testing, *in vitro* efficacy, and safety assessments of hAMSCs, including their ability to secrete cytokines and regulate immunity. In addition, *in vitro* and *in vivo* tumorigenicity experiments, acute and long-term toxicity evaluations in animal experiments, immunotoxicity experiments, and *in vitro* genetic analysis were carried out. After passing these evaluations, the rescue treatment with hAMSCs was initiated. This decision was based on the literature on intravenous/local injection of hAMSCs and the maximum tolerated dose determined from animal experiments (Jiao et al., 2016; Gao et al., 2020; Qin et al., 2022). The treatment protocol included intravenous injection of hAMSCs (1.0 × 10^6^ cells/kg), local muscle injection along the edge of the skin wound (2.0 × 10^4^ cells/cm^2^), and local application of stem cell culture supernatant on the wound, with adjustments made in line with the wound and the general condition of the patient. Preclinical research on hAMSCs and regenerative treatments for CUA patients was illustrated in our previous work (Supplementary Figure S1; Qin et al., 2022; Wang et al., 2022a; Bian et al., 2023; Lu et al., 2025).

In a patient with CUA undergoing hAMSC treatment, the healing progress of skin lesions and the histological restoration of skin integrity were illustrated in our previous work (Supplementary Figures S2 and S3; Qin et al., 2022). *In vitro* studies have shown that co-culturing hAMSCs with peripheral blood mononuclear cells from healthy controls depends on the cytokines contained in their supernatant, regulating the proliferation of T cells and lymphocytes (Qin et al., 2022). In a CUA patient, severe skin damage with ulcers and eschar formation, along with elevated levels of blood leukocytes, C-reactive protein, and Th1/Th2 ratios, was observed before treatment. After 15 months of hAMSC therapy, the skin wounds of the patient healed, and the levels of blood leukocytes, C-reactive protein, and Th1/Th2 decreased (Wang et al., 2022a). Immune cell subset analysis showed that hAMSCs were able to inhibit the proliferation and differentiation of Th1/Th17 cells, induce the proliferation of functional Treg cells with anti-inflammatory effects, and regulate immune function. In addition, before treatment, the patient exhibited significantly elevated platelets, D-dimer, and fibrinogen levels. After 1 week of treatment, a decrease in the levels of these markers was observed. Furthermore, after 3 months of treatment, platelet and fibrinogen levels approached normal, with D-dimer gradually decreasing to physiological levels after 6 months (Bian et al., 2023).

### The rationale for MSC treatment in calciphylaxis and the superiority of hAMSCs

Table 4 summarizes preclinical and clinical studies of MSCs for the treatment of CUA and related diseases. hAMSCs represent a paradigm shift in regenerative medicine, offering a safe, efficacious, and ethically sound alternative to conventional MSCs. Their unique immunomodulatory profile, combined with robust paracrine activity, positions them as a frontline therapy for calciphylaxis and other inflammatory, ischemic, and critical disorders. Our team is conducting in-depth research on the treatment of calciphylaxis using hAMSCs (NCT04592640).

**Table 4:** Preclinical and clinical studies of MSCs for calciphylaxis and related diseases.

	Research object	Diseases	MSC type	Reference
Preclinical study	C57BL/6 mice	Acute lung injury	Human ADMSCs	Wang et al. (2020b)
	C57BL/6 mice	Myocardial ischemia–reperfusion injury	HUMSCs	Hou et al. (2024a)
	Wistar rats	Bone defect	HUMSCs	Zhang et al. (2021)
	C57BL/6 mice	Full-thickness skin defect	Human ADMSCs	Pi et al. (2022)
	Wistar rats	Disseminated intravascular coagulation	Rat BMSCs	Wang et al. (2017)
	C57BL/6 mice	CKD	Mouse BMSCs	Wei et al. (2021)
	Sprague Dawley rats	CKD	BMSCs	Liu et al. (2022a)
	Sprague Dawley and Lewis rats	CKD	Mouse ADMSCs	Yokote et al. (2017)
	MRL/lpr mice	Systemic lupus erythematosus	HUMSCs	Cai et al. (2015)
	C57BL/6 mice	Thermal burn skin wound	hAMSCs	Li et al. (2019)
	C57BL/6 mice	Chronic iron-overload wound	Human ABCB5^+^ dermal MSCs	Vander Beken et al. (2019)
	Sprague Dawley rats	Diabetic wound	HUMSCs	Wang et al. (2024a)
	Sprague Dawley rats	Diabetic ulcers	Rat ADMSCs	Liang et al. (2024)
	BKS.Cg-m^+/+^Leprdb/J mice	Diabetic ulcers	Human ADMSCs	De Gregorio et al. (2020)
	C57BL/6 mice	Diabetic ulcers	Mouse BMSCs	Zhou et al. (2024)
Clinical study		Systemic lupus erythematosus	HUMSCs	Yuan et al. (2019)
		COVID-19	Human BMSCs	Zhu et al. (2021)
		Severe limb ischemia	HPMSCs	Wang et al. (2021)
		Diabetic ulcers	HUMSCs	Zhang et al. (2022)
		Diabetic ulcers	Human ABCB5^+^ dermal MSCs	Kerstan et al. (2022)
		Calciphylaxis	hAMSCs	Qin et al. (2022)

## Future research recommendations

**Figure 5:**
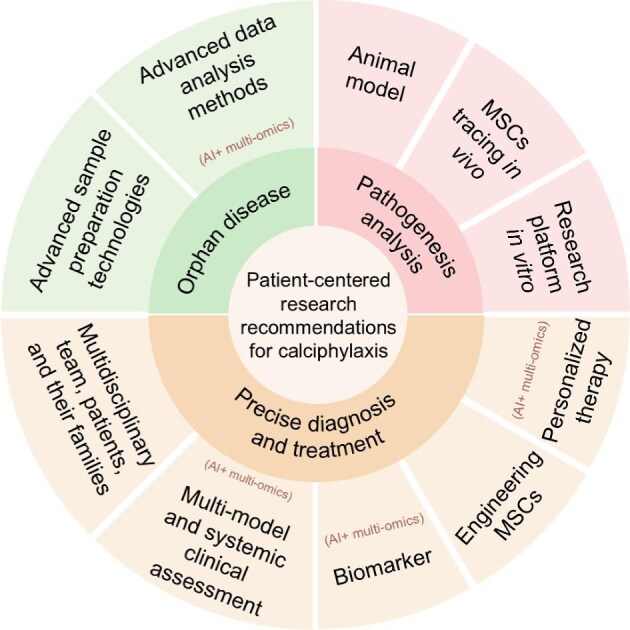
Future research recommendations for calciphylaxis.

The recommended future research is presented in Figure 5.

### Advanced technologies rooted in an orphaned background

Given the low prevalence of calciphylaxis and the occurrence of NUC, along with the challenges of misdiagnosis and underdiagnosis, advanced technologies are necessary to analyze the pathogenesis of this rare disease, utilizing small human sample sizes and minimal tissue amounts. The limited availability of tissue necessitates the use of more sophisticated sample preparation techniques, such as single-cell or spatial omics. Moreover, the small sample size requires the integration of advanced analytical methods, including AI-based approaches for time-series or multimodal analyses.

### Highly simulated preclinical research models

The specific pathogenesis of calciphylaxis remains unclear due to the lack of preclinical research models. Developing animal models or *in vitro* research platforms that accurately replicate calciphylaxis is crucial for identifying biomarkers and validating the rescue mechanisms of MSCs. Implementing *in vivo* tracer technology for MSCs can help elucidate their role in tissue repair and the underlying therapy mechanisms.

### Patient-centered multidisciplinary collaboration

Managing calciphylaxis is complex and lengthy, requiring a multidisciplinary team and a robust doctor–patient relationship. Patients and their families play active roles throughout the entire research cycle.

### Precise diagnosis and noninvasive biomarkers

Skin histopathology, although considered the gold standard for diagnosis, is invasive and should be integrated with patient history, clinical manifestations, hematological indicators, and imaging examinations. The use of noninvasive biomarkers, multimodal and systemic assessments, enhanced by AI technologies, improves diagnostic accuracy and deepens the understanding of CUA and NUC.

### Novel, effective, economical, and tailored treatments

Due to the lack of large-scale prospective studies, no therapeutic agents have been approved. Racial and individual heterogeneity may limit the efficacy of conventional treatments. There is an urgent need for effective, accessible, and cost-effective therapies. It is recommended to evaluate biomarkers for efficacy and prognosis in calciphylaxis patients treated with MSCs using multi-omics techniques. Engineering MSCs for precision-targeted therapy is promising to enhance therapeutic efficacy.

## Conclusion

Calciphylaxis is a rare progressive disease characterized by subcutaneous adipose and dermal microvascular lesions, with rapid progression and high mortality due to infection. However, vascular calcification cannot completely explain its dangerous prognosis. The induction of severe ischemia and inflammatory responses has been emphasized. Monotherapy for calciphylaxis poses challenges. Safe, effective, accessible, and economical MSC strategies guided by noninvasive biomarkers have promising therapeutic potential due to their ability to inhibit vascular calcification, exhibit anti-inflammatory and immune-modulating effects, improve hypercoagulability, and promote tissue repair.

## Supplementary Material

mjaf009_Supplemental_File

## Notes

**Author contributions:** N.W., D.W., and S.L. conceived the manuscript. D.W., S.L., J.H., C.L., M.Z., J.W., X.T., and T.L. wrote the draft and N.W., Y.Z., J.L., and L.Q. revised the manuscript. S.L., J.H., and T.L. drew the images. X.T. helped obtain the patient's clinical data and revise the images. All authors contributed substantially to the discussion of the content and edited the manuscript before submission.
