# Supplementary material for: Calciphylaxis: ongoing challenges and treatment opportunities with mesenchymal stem cells
Source: J Mol Cell Biol. 2025 Mar 17;17(2):mjaf009. doi: 10.1093/jmcb/mjaf009 (PMC12405897; doi:10.1093/jmcb/mjaf009)
Supplement: mjaf009_Supplemental_File [file mjaf009_supplemental_file.pdf]

Supplementary Material

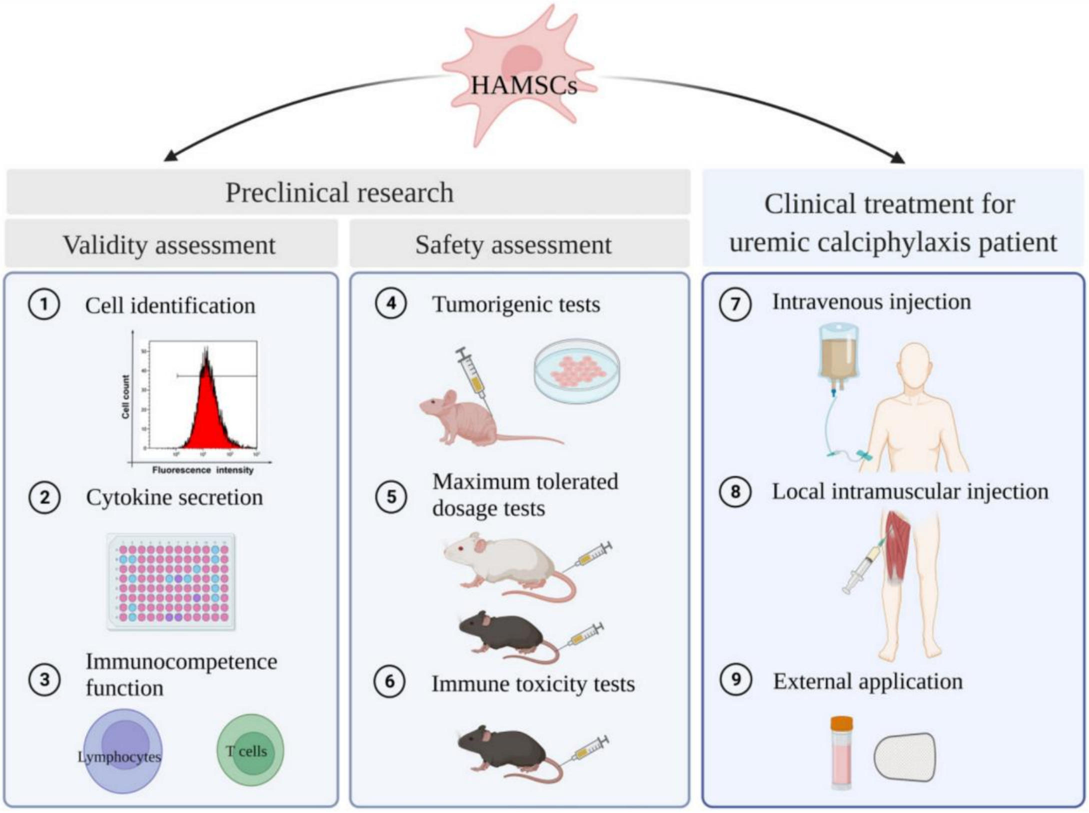

Figure S1. Preclinical research of hAMSCs and regenerative treatment for the uremic calciphylaxis patient[1].

hAMSC, human amnion-derived mesenchymal stem cell.

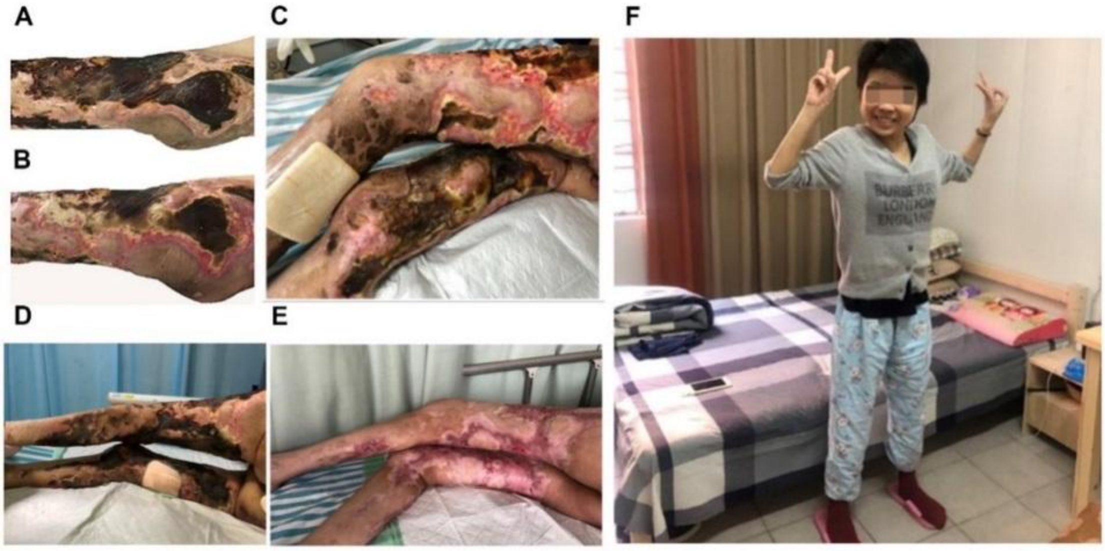

Figure S2. Clinical hAMSC treatment for the uremic calciphylaxis patient[1].

(A) Wounds on the lower limbs before hAMSC treatment. (B) After 14 days of intravenous infusion plus local intramuscular injection, the surface of wounds began to recover. (C) The left thigh with local intramuscular hAMSC injection for 1 month recovered better than the right thigh without local intramuscular injection. (D) Wounds of lower limbs before treatment. (E) Skin lesions were healed after treated with hAMSCs for 1 year. (F) The patient could walk after 15 months of hAMSC treatment.

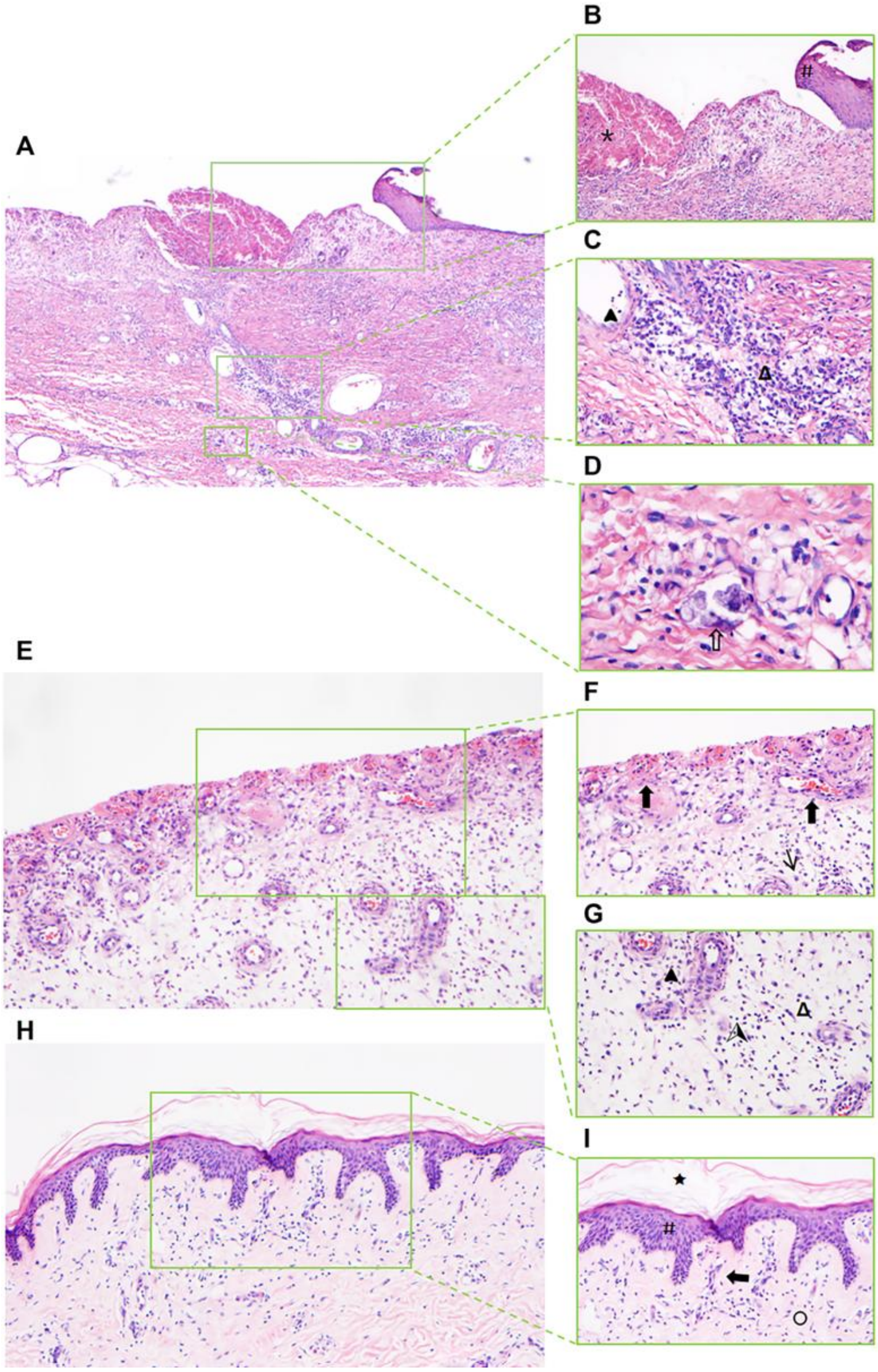

Figure S3. H&E staining of skin biopsy from the calciphylaxis patient during hAMSC treatment[1].

(A–D) Specimens of biopsy obtained from the margin of an ulcer on the thigh before hAMSC treatment. (A) Pathological characteristics contain exfoliation of epidermis, necrosis, inflammation, and vascular calcification (magnification, 40×). (B) Exfoliation of epidermis (#), necrosis (*), and marked inflammatory response (magnification, 100×). (C) Extensive infiltration of inflammatory cells mainly presented as plasmacytes (△) and neutrophils (▲) (magnification, 200×). (D) Sheet-like

calcium deposits on the wall of small vessels (⇧) characterieed as granular basophilic plauue (400×). (E−G) Skin biopsy from the thigh of the calciphylaxis patient after hAMSC treatment for 1 month. (E) Pathological characteristics contain nascent granulation tissue, reduced inflammatory response, and no epidermal tissue (magnification,100×). (F) Proliferation of myofibroblasts (↑) and regeneration of blood vessels (⬆) (magnification,200×). (G) Reduced plasmacytes (Δ), neutrophils (▲), and lymphocytes (⅄) in the subcutaneous tissue (magnification, 200×). (H and I) Skin biopsy from the thigh of the calciphylaxis patient after hAMSC treatment for 20 months. (H) Pathological characteristics contain regeneration of epidermal and dermal layers, mature vessels without calcification, collagen remodeling, and mild inflammation (magnification, 100×). (I) Intact cuticle (★), restoration of damaged epidermis integrity (#), collagen fiber (○), and fewer inflammatory cells (magnification, 200×)

References

[1] QIN L, ZHANG J, XIAO Y, LIU K, CUI Y, XU F, REN W, YUAN Y, JIANG C, NING S, YE X, ZENG M, QIAN H, BIAN A, LI F, YANG G, TANG S, ZHANG Z, DAI J, GUO J, WANG Q, SUN B, GE Y, OUYANG C, XU X, WANG J, HUANG Y, CUI H, ZHOU J, WANG M, SU Z, LU Y, WU D, SHI J, LIU W, DONG L, PAN Y, ZHAO B, CUI Y, GAO X, GAO Z, MA X, CHEN A, WANG J, CAO M, CUI Q, CHEN L, CHEN F, YU Y, JI Q, ZHANG Z, GU M, ZHUANG X, LV X, WANG H, PAN Y, WANG L, XU X, ZHAO J, WANG X, LIU C, LIANG N, XING C, LIU J, WANG N. A novel long-term intravenous combined with local treatment with human amnion-derived mesenchymal stem cells for a multidisciplinary rescued uremic calciphylaxis patient and the underlying mechanism. J Mol Cell Biol, 2022, 14(2): mjac010.
